# Abdominal Apoplexy: A Case Study of Idiopathic Spontaneous Lesser Sac Hematoma

**DOI:** 10.7759/cureus.4937

**Published:** 2019-06-18

**Authors:** Ulugbek Negmadjanov, Levonti Ohanisian, David Rubay, Boris Hristov, Avraham Belizon

**Affiliations:** 1 Surgery, Charles E. Schmidt College of Medicine, Florida Atlantic University, Boca Raton, USA; 2 Orthopaedic Surgery, Charles E. Schmidt College of Medicine, Florida Atlantic University, Boca Raton, USA; 3 Surgery, Charles E. Schmidt College of Medicine at Florida Atlantic University, Boca Raton, USA

**Keywords:** idiopathic spontaneous intraabdominal hemorrhage, abdominal apoplexy, middle colic artery

## Abstract

Idiopathic spontaneous intraperitoneal hemorrhage (ISIH) is a rare event associated with high mortality. There have been multiple case reports of spontaneous rupture of middle colic pseudoaneurysms in the literature. Herein, we present a case of a 51-year-old female that presented with spontaneous rupture of the middle colic artery and associated massive intraabdominal hematoma without findings of a pseudoaneurysm. The patient underwent a computed tomography (CT) scan as an outpatient 24 hours prior to the onset of the bleeding due to abdominal pain without findings of hematoma or aneurysm of the mesenteric vessels. Subsequently, the patient underwent emergent exploratory laparotomy with findings of a massive hematoma in the lesser sac and spontaneous bleeding from the middle colic artery that was ligated. The patient had an uneventful postoperative course and fully recovered. To our knowledge, this is the second reported case of idiopathic bleeding from the middle colic artery without evidence of a pseudoaneurysm based on a current review of the literature.

## Introduction

Idiopathic spontaneous intraperitoneal hemorrhage (ISIH), also referred to as abdominal apoplexy, is a rare and potentially fatal condition [1] that is often seen in elderly hypertensive patients with atherosclerosis [2]. It can also result from congenital vascular defects in younger patients, especially young women, who are prone to rupture during pregnancy. Clinical presentation varies from nonspecific abdominal pain, nausea, and vomiting, to an acute abdomen with cardiovascular collapse [3]. Preoperative diagnosis sometimes can be confirmed by angiography, but often patients are diagnosed during surgical exploration. Therefore, a high index of suspicion should be maintained and the diagnosis of ISIH should be included in the differential diagnosis of any unexplained intra-abdominal hemorrhage [4], as early operation with ligation of the bleeding vessel offers an excellent chance for recovery [5].

## Case presentation

**Figure 1:**
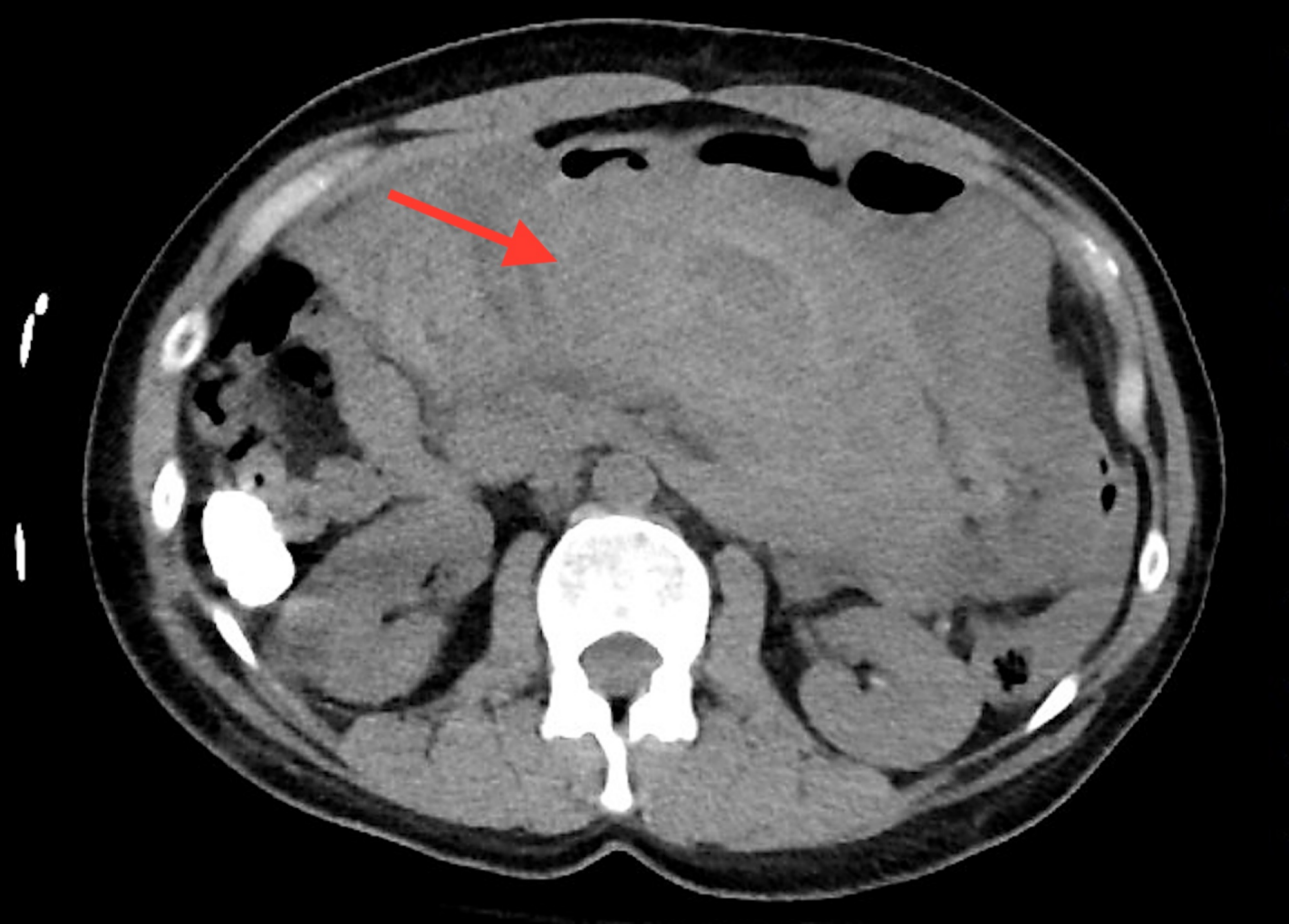
Axial view demonstrating a mixed density collection around the region of the pancreas and adjacent mesentery

**Figure 2:**
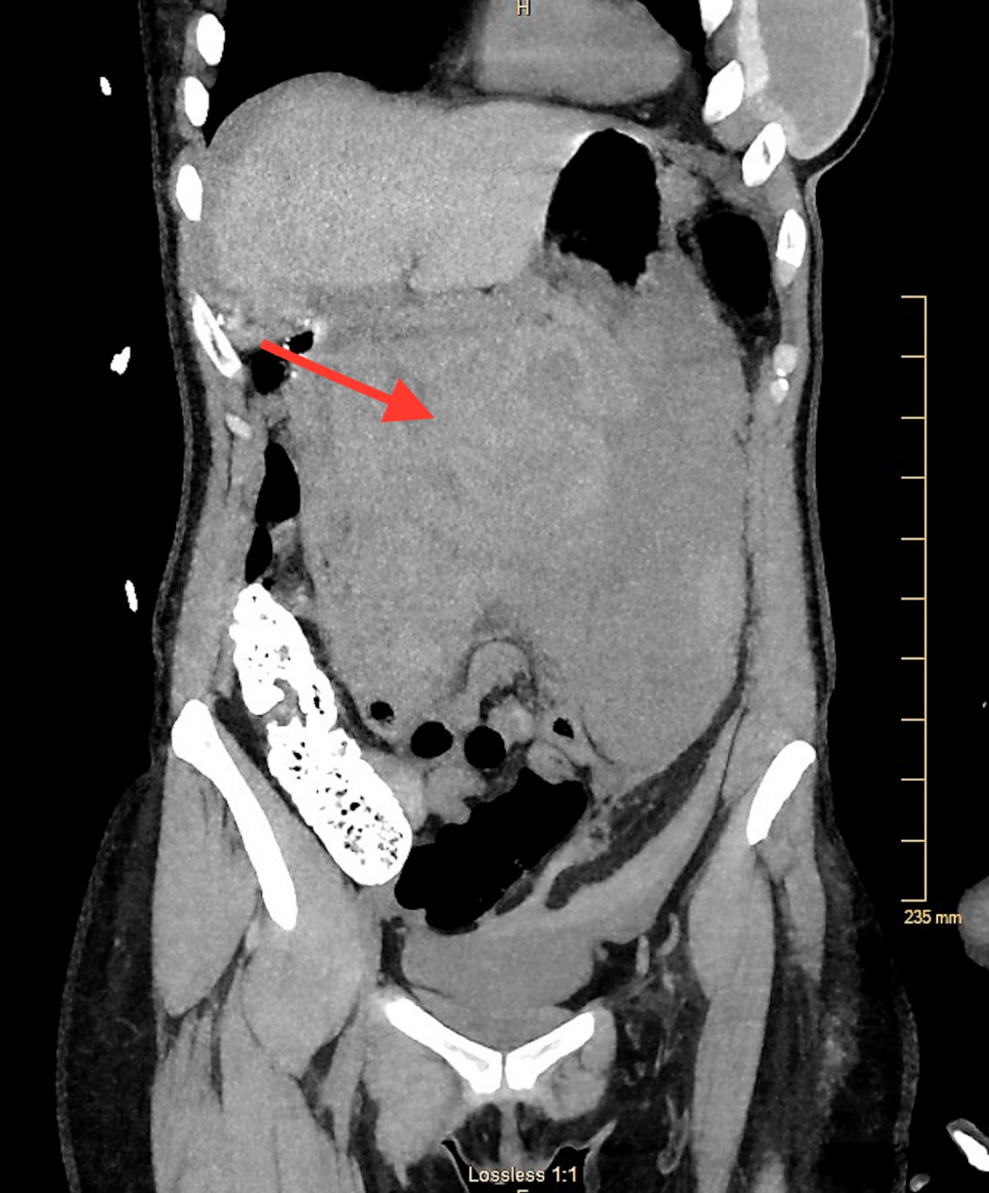
Coronal view demonstrating a mixed density collection around the region of the pancreas and adjacent mesentery

The patient is a 51-year-old female who presented to the emergency department with the sudden onset of generalized abdominal pain and distention. She had also reportedly fainted at home, according to a family member. The patient denied any recent trauma, however, she did mention diarrhea for the past 24 hours and vague abdominal pain for one week prior to the episode. The patient reported being seen earlier in the day by her primary care provider, and that she had a computed tomography (CT) scan of the abdomen and pelvis with intravenous contrast done as part of her workup, with findings of a mildly dilated stomach and no findings of an intra-abdominal hematoma. Her past medical history was notable for breast augmentation, but no history of vasculitis, connective tissue disorder, or hypertension was noted. On admission, she was hypotensive, with a blood pressure of 85/40, a pulse of 98, and a body temperature of 96.3°F. On physical examination, her abdomen was distended and tenderness was diffusely present on palpation. Her complete blood count results were as follows: white blood cell count 19 × 109/L, hemoglobin 10.4 g/dL, and hematocrit 30.9%. The prothrombin time, partial thromboplastin time, platelets, liver function tests, amylase, and lipase were within normal limits. The patient received crystalloid resuscitation and underwent a CT scan of the abdomen and pelvis without contrast, which demonstrated a 6 cm x 15 cm mixed density collection around the region of the pancreas and adjacent mesentery, displacing the stomach anteriorly (Figure 1, Figure 2).

 At that time, the patient was taken for urgent exploratory laparotomy. During laparotomy, approximately 2 liters of free blood and clots were found in the abdomen, emanating from the lesser sac. The lesser sac was entered by dividing the gastrocolic ligament and arterial bleeding from the middle colic artery at the root of mesentery of the transverse colon was identified. The artery was subsequently ligated without a colectomy since the blood supply to the transverse colon had not been compromised. Further inspection, including duodenal Kocherization, an inspection of the lesser sac, pancreas, spleen, liver, and pelvis, did not identify any other sources of bleeding. The patient's postoperative course was uneventful, and she was discharged home on the sixth postoperative day. Her follow-up visits were unremarkable.

## Discussion

ISIH has been classically used to describe cases of intraperitoneal bleeding from visceral vessels that are not a consequence of typical causes of hemorrhage, including trauma, iatrogenic injury, vascular diseases, visceral malignancy, or ectopic pregnancy, or after hemorrhage from a grossly apparent aortic aneurysm or aortic dissection is excluded [6]. Visceral vessel rupture can often occur at the site of an aneurysm, the most common sites being the splenic artery (60%), hepatic artery (20%), superior mesenteric artery (5.5%), celiac artery, gastrointestinal/epiploic arteries (4%), intestinal artery (3%), and duodenal/pancreatic arteries (1.5%). Moreover, only 2% of all reported splanchnic artery aneurysms have been located in the jejunal, ileal, or colic arteries [2].

ISIH is synonymous with the more recent term “abdominal apoplexy,” which has been used to describe cases of intra-abdominal bleeding that are not a consequence of numerous well-documented causes such as trauma, pregnancy, vasculitis, malignancy, or inflammatory processes (e.g. pancreatitis) [7]. Considering that colic artery aneurysms represent approximately 0.28% [5] of all superior mesenteric aneurysms, the rupture of a middle colic artery aneurysm is a particularly rare cause of abdominal apoplexy, with only 35 cases reported prior to our case [5-6].

The treatment of idiopathic spontaneous intra-abdominal hemorrhage revolves around patient resuscitation and management of the source of bleeding. In case of a ruptured aneurysm of the middle colic artery, surgical management includes an emergency laparotomy, arterial ligation, and resection of the aneurysm. Endovascular embolization has been suggested as a safe and less invasive alternative approach. To our knowledge, this is the second reported case of idiopathic bleeding from the middle colic artery without evidence of a pseudoaneurysm, based on a current literature review [7].

## Conclusions

Idiopathic spontaneous intraperitoneal hemorrhage (ISIH), also referred to as abdominal apoplexy, is a rare and potentially fatal condition whose etiology remains unclear in most cases. We present the case of a 51-year-old female with ISIH that, to our knowledge, is the second such case without evidence of a pseudoaneurysm, based on a current literature review. We hope that by presenting this case, we may add to the body of literature to further elucidate improved methods of diagnosis and early recognition and to reduce morbidity in future cases.
